# Hemodynamic Response to Lipopolysaccharide Infusion and Effect of Meloxicam Administration on Cardiac Function in Donkeys

**DOI:** 10.3390/ani14243660

**Published:** 2024-12-18

**Authors:** Francisco J. Mendoza, Antonio Buzon-Cuevas, Raul Aguilera-Aguilera, Carlos A. Gonzalez-De Cara, Adelaida De Las Heras, Alejandro Perez-Ecija

**Affiliations:** 1 Department of Animal Medicine and Surgery, University of Cordoba, 14014 Cordoba, Spainalejandro.perez.ecija@uco.es (A.P.-E.); 2 Egabro Veterinary Center, Cabra, 14940 Cordoba, Spain; vet.raulaguilera@gmail.com

**Keywords:** Andalusian donkeys, arterial pressure, cardiac output, central venous pressure, echocardiography, endotoxemia treatment

## Abstract

Systemic inflammatory response syndrome (SIRS) in donkeys is observed to be secondary to colic, diarrhea or pleuropneumonia, among other disorders. Horses with SIRS develop secondary disturbances such as hyperlipemia, laminitis, disseminated intravascular coagulopathy, and hemodynamic and cardiac derangements, which impair their prognosis and increase the mortality rate. In donkeys, no information is available on the effect of experimentally induced endotoxemia in the cardiovascular system. Acute experimental endotoxemia was induced by lipopolysaccharide (LPS) infusion in six healthy adult non-pregnant jennies. Physical signs, arterial (systolic, diastolic and mean) and central venous pressure were monitored during 360 min. Cardiac troponin I (cTnI) concentrations were measured in blood samples, and echocardiography was performed. LPS infusion caused an increase in cTnI, hypotension and diminution of central venous pressure, cardiac dysfunction, with a decrease in stroke volume (SV), cardiac output (CO) and cardiac index, and impairment of ultrasonographic ventricular function parameters. Intravenous meloxicam administration prevented the cTnI increase, hypotension, diminution of SV and CO, and changes in ultrasonographic parameters related to ventricular dysfunction. Thus, meloxicam could be proposed as an effective therapeutical option to control the hemodynamic and cardiac derangements observed in donkeys with SIRS.

## Simple Summary

Systemic inflammatory response syndrome (SIRS) in donkeys is observed to be secondary to colic, diarrhea or pleuropneumonia, among other disturbances. Horses with SIRS have hemodynamic and cardiac derangements, impairing prognosis and increasing mortality, but no information is available in donkeys with SIRS. Lipopolysaccharide (LPS) infusion in healthy adult donkeys led to increased cardiac troponin I concentrations (cTnI), hemodynamic derangements such as hypotension and diminution of central venous pressure, as well as cardiac dysfunction with decrease in stroke volume, cardiac output and cardiac index, and impairment of ultrasonographic ventricular function parameters. Intravenous meloxicam administration prevented most of the hemodynamic and cardiac deleterious effects of LPS infusion.

## 1. Introduction

Donkey medicine is a growing area of interest for clinicians, researchers and owners worldwide [1]. Although many breeds are in a serious danger of extinction, donkeys still maintain their role as a workforce in developing countries and are also increasingly demanded in other fields such as assisted-therapy, pet animals, novel equid sports and show events, or as a source of nutritional by-products [2,3]. Since differences between donkeys and horses are plentiful, extrapolating information between species could lead to misdiagnoses, unnecessary treatments and increased veterinary expenses [4,5,6,7]. Thus, donkey-specific information is needed for a specialized veterinary care, which is currently in high demand.

Systemic inflammatory response syndrome (SIRS) is observed in donkeys secondary to similar disturbances in horses, such as colic, diarrhea, and pleuropneumonia, among other diseases [8]. SIRS-related complications described in horses, such as laminitis, hyperlipemia, disseminated intravascular coagulation, hemodynamic and cardiac disturbances and multiorgan dysfunction [9] can also be seen in donkeys with SIRS [10,11,12]. Contrary to horses [13,14,15], information on the pathogenesis, complications and therapeutical management of SIRS is scarce in donkeys [16,17]. Recently, our research group studied the effects of experimentally induced endotoxemia on acute-phase protein concentrations, blood storage conditions, and the expression and concentration of inflammatory cytokines in donkeys [18,19,20].

In horses, experimentally induced endotoxemia induces an increase in cardiac troponin concentrations [21], demonstrating direct damage in the myocardium. Moreover, experimentally induced endotoxemia modifies the cardiac function, causing deleterious hemodynamic effects such as hypotension and a decreased systemic vascular resistance [22,23]. To the authors’ knowledge, there are no studies evaluating the effect of SIRS on the cardiovascular system in donkeys. Moreover, reference ranges for high-sensitive cardiac troponin I (cTnI), which is considered the laboratory gold standard for the diagnosis of myocardial damage [24], are not available in healthy donkeys.

Although multiple therapeutical options for SIRS treatment have been evaluated in horses [25,26,27,28,29], only flunixin meglumine [17] and meloxicam [18] have been studied in lipopolysaccharide (LPS)-challenged donkeys. The pharmacokinetics of meloxicam in healthy donkeys is different to that of horses, with a lower mean residence and distribution times, and a faster clearance than that of horses [30].

We hypothesize that experimentally induced endotoxemia will cause deleterious changes in blood pressure and cardiac parameters, and that meloxicam could avoid these effects. Thus, the objectives of this study were the following: (a) to establish blood reference ranges for cTnI in healthy donkeys; (b) to evaluate the effect of acute experimentally-induced endotoxemia on blood cTnI in donkeys; (c) to study the effect of endotoxemia on blood pressure and cardiac function, and (d) to assess if meloxicam administration can prevent the effects of experimentally induced endotoxemia on cTnI, blood pressure and cardiac function in donkeys.

## 2. Materials and Methods

### 2.1. High-Sensitive cTnI Characterization

Blood samples were collected from 68 healthy (6.7 ± 0.6 years old; 9 jacks and 59 jennies) Andalusian and Andalusian cross-bred donkeys from two different farms with similar premises in southern Spain. Animals were considered healthy based on normal anamnesis and physical examination, a normal blood analysis, and no treatment administration for at least 2 months before sampling.

Blood samples were collected by venipuncture from the jugular vein into plain tubes with clot activator and kept on ice until centrifugation; aliquots were frozen at −20 °C until measurement. Serum samples were sent on dry ice to IDEXX Laboratories Inc.(Barcelona, Spain) for high-sensitive cardiac troponin I measurements (Advia Centaur TnI-Ultra assay; Siemens Healthcare Diagnostics, Barcelona, Spain). The detection limit was 0.006 ng/mL, and a concentration of 0 ng/mL was assigned to animals with concentrations below detection.

### 2.2. Experimental Design

#### 2.2.1. Endotoxemia Induction

Endotoxemia was induced in six healthy adult (7.6 ± 0.8 years old) Andalusian nonpregnant jennies (348 ± 39 Kg), through intravenous infusion of a 20 ng/Kg dose of LPS from *E. coli* O55:B55 over 30 min (from −30 to 0 min), as previously reported [18]. Donkeys were randomly assigned to receive either an intravenous bolus of 20 mL of saline solution (control group) or 0.6 mg/Kg of intravenous meloxicam (Loxicom, Norbrook, Northern Ireland, UK; meloxicam group) when LPS infusion was finished (time 0). Two experiments were performed on each animal with a one-month washout period between them.

Blood samples were drawn at −30, 0, 30, 60, 90, 120, 150, 180, 240 and 360 min post-LPS from a jugular catheter into K_3_-EDTA tubes to determine leukocyte and neutrophil counts (Lasercyte, IDEXX Laboratories Inc., Hoofddorp, the Netherlands), and into heparin tubes to measure plasma tumor necrosis factor alpha (TNFα) concentrations [18]. SIRS was established when at least two of the following criteria were met: tachycardia, tachypnea, fever, and leukopenia and neutropenia [31].

#### 2.2.2. Animal Monitoring for Hemodynamic Evaluation

Donkeys were monitored during the entire experiment (from −30 to 360 min post-LPS) using a multiparametric monitor (VetCare, BBraun, Barcelona, Spain). The following parameters were obtained with this equipment: rectal temperature (rectal probe), heart rate and electrocardiogram (ECG) using a base–apex lead; oscillometric non-invasive blood pressure (NIBP), with data from systolic (SAP), diastolic (DAP) and mean (MAP) arterial pressures, using a cuff bladder placed in the proximal tail base and centered over the coccygeal artery with a width-to-tail girth ratio of 0.4–0.6 (manufacturer’s recommendations); and mean central venous pressure (CVP) using a sterile catheter (19 G 90 cm long line catheter, Mila International Inc., Florence, KY, USA) introduced aseptically in the right jugular vein up to the right atrium or distal vena cava. Once the catheter’s tip location was confirmed, based on waveform characteristics, the transducer was positioned at shoulder height and adjusted to measure 0 mmHg at atmospheric pressure. Three non-consecutive measurements were recorded at each time.

In addition, heart and respiratory rates (HR and RR, respectively), gastrointestinal motility (four quadrants), mucous membrane color (MMC), presence of toxic line, capillary refill time (CRT), and digital pulse were measured by the operators, each 15 min from −30 to 240 min post-LPS, and then at 360 min post-LPS infusion.

Systemic vascular resistance (SVR) was calculated using a formula previously described in horses [22]:

SVR = (MAP − CVP)/Cardiac Output (CO) × 80

#### 2.2.3. Cardiac Function in Experimentally Induced Endotoxemic Donkeys

Blood samples were collected from a jugular catheter for serum cTnI measurements at −30, 0, 60, 90, 120, 180 and 240 min post-LPS infusion. Sample preparation and measurements were conducted as described above.

Transthoracic echocardiography (MyLab 50 XVision machine with a 2.5–3.5 MHz phased-array sector transducer; Esaote, Barcelona, Spain) was performed every 30 min (from −30 to 240 min post-LPS infusion), by the same operator (FJM) using the right parasternal imaging planes in D-mode and M-mode (long- and short-axis views), following a standardized protocol previously described for donkeys and horses [32,33,34,35]. A base–apex lead ECG was recorded for timing within the cardiac cycle. In each plane, at least five non-consecutive representative cardiac cycles were recorded. All images were stored either as 2D cine-loops (D-mode) or as still images (M-mode) in digital raw-data format for later analysis.

All cardiac measurements were performed offline by a blinded observer (ABC) using a dedicated analysis software package (MyLab™ Desk, Esaote, Spain), and following protocols previously described for donkeys and horses [32,33,34,35]. End-diastolic measurements were made at the peak of the ECG R wave, whereas end-systolic measurements were taken at the point of maximal excursion of the interventricular septum and the left ventricular free wall. When available recordings did not contain enough complete cardiac cycles for all imaging planes, only available cycles were measured. Furthermore, when unmistakable identification of anatomical landmarks was not possible, only cardiac cycles with clear landmarks were considered. The HR of each cycle was collected from the ultrasound machine ECG, and if any discordance was detected, the HR was checked with the recorded HR from the multiparametric monitor and manual HR. The cardiac index (CI) was calculated using the formula previously described in horses [36]: CI = CO/body surface area (BSA). Descriptions of measured and calculated parameters are compiled in Supplementary Tables S1 and S2.

### 2.3. Data Analysis

Normality was assessed using the Shapiro–Wilk test. Normally distributed data are expressed as the mean and standard error of the mean (SEM), and those that were not normally distributed are expressed as median and interquartile range (IQR, 25th–75th percentile). Percentiles were calculated using the Tukey’s Hinges test. Differences between groups were determined using either a Mann–Whitney or unpaired T-student test, according to normality. Differences among time-points within each group were studied using an ANOVA of repetitive measures followed by a Bonferroni post hoc analysis or a Friedman test followed by a Dunn analysis. A *p* value <0.05 was considered significant. Reference ranges were obtained with a robust method, using dedicated software (Reference Value Advisor v. 2.1. freeware, available at: http://www.biostat.envt.fr/reference-value-advisor/ (accessed on 7 October 2024)), as recommended by the American Society of Veterinary Clinical Pathology [37]. Statistical analysis was performed using a commercial statistical software (IBM SPSS Statistics 28, IBM, Chicago, IL, USA).

## 3. Results

### 3.1. High-Sensitive cTnI Characterization

The mean ± standard error serum cTnI concentration in healthy donkeys was 0.012 ± 0.002 ng/mL, with a median of 0 and an IQR of 0.02, with a 95% CI of the mean between 0.0071 and 0.0158 ng/mL. Reference ranges were between 0 and 0.047 ng/mL. In 34 donkeys, the cTnI concentration was below the detection limit.

### 3.2. Effect of LPS Infusion

All donkeys developed typical features of SIRS such as tachycardia, fever, leukopenia and neutropenia, and an increase in plasma TNFα concentration from 30 min post-LPS infusion (data previously published) [18]. Meloxicam administration attenuated the increases in heart rate and plasma TNFα concentrations (data previously published) [18].

### 3.3. Effect of Experimentally Induced Endotoxemia and Meloxicam Administration on cTnI

**Figure 1:**
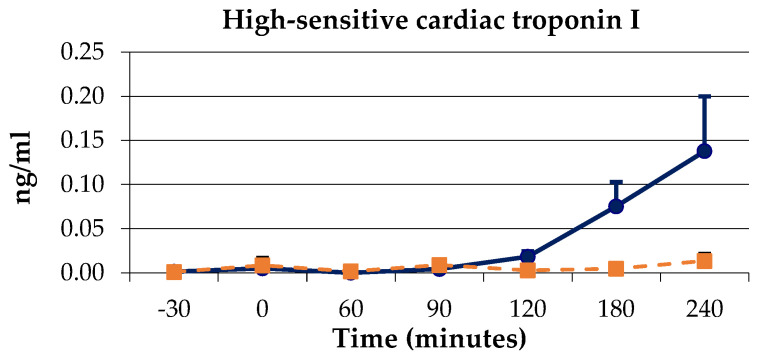
High-sensitive cardiac troponin I concentrations in donkeys with acute experimentally-induced endotoxemia receiving either saline solution (control group, blue line) or meloxicam (treated group, orange line).

LPS infusion induced a significant (*p* < 0.05) increase in serum cTnI concentrations compared to baseline from 120 min post-LPS until the end of the experiment (Figure 1). Meloxicam administration prevented the increase in cTnI concentrations (Figure 1).

### 3.4. Effect of Experimentally Induced Endotoxemia and Meloxicam Administration on Hemodynamic Parameters

LPS infusion induced a significant (*p* < 0.05) increase in HR from 0 until 195 min post-LPS. Meloxicam avoided the appearance of tachycardia.

**Figure 2:**
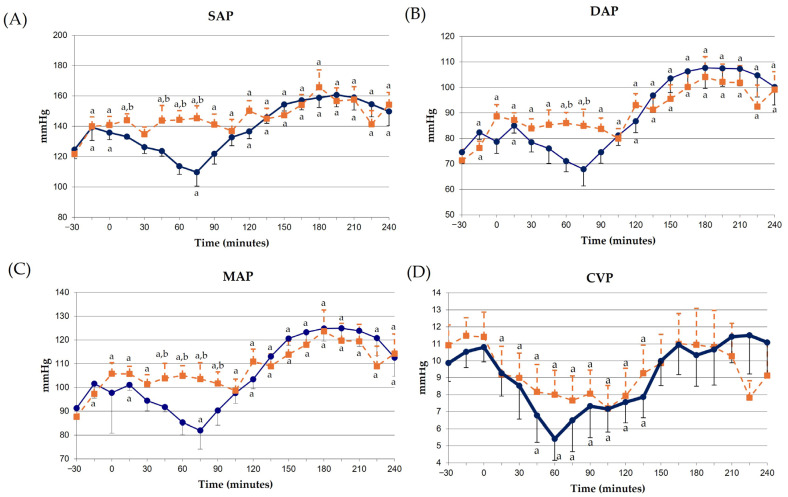
Arterial blood pressures: (**A**) systolic (SAP), (**B**) diastolic (DAP), and (**C**) mean (MAP) and (**D**) central venous pressure (CVP) in donkeys with acute experimentally-induced endotoxemia receiving either saline solution (control group, blue line) or meloxicam (treated group, orange line). ^a^ *p* < 0.05 vs. baseline; ^b^ *p* < 0.05 vs. control group.

**Figure 3:**
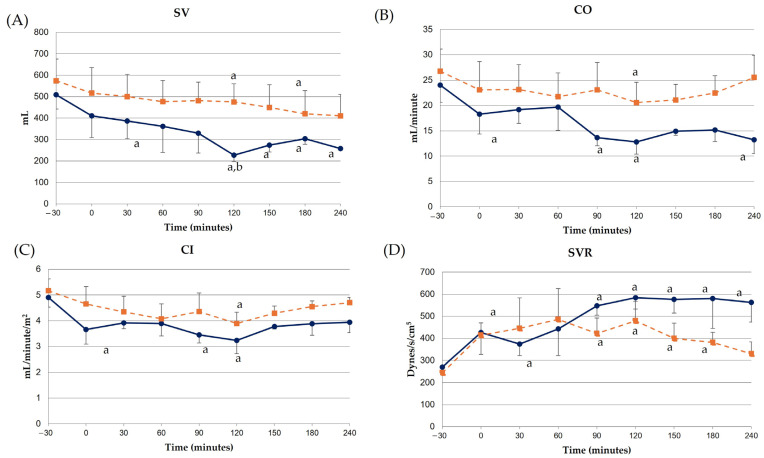
(**A**) Stroke volume (SV), (**B**) cardiac output (CO), (**C**) cardiac index (CI) and (**D**) systemic vascular resistance (SVR) in donkeys with acute experimentally-induced endotoxemia receiving either saline solution (control group, blue line) or meloxicam (treated group, orange line). ^a^ *p* < 0.05 vs. baseline; ^b^ *p* < 0.05 vs. control group.

SAP, DAP, and MAP decreased in response to LPS infusion from 0 to 60–75 min post-LPS, showing a later rebound effect and remaining above baseline until the end of the experiment (Figure 2A–C). CVP also decreased from 0 min, but remained below baseline until 150 min post-LPS (Figure 2D). SVR remained above baseline from 0 min until the end of the experiment (Figure 3D).

Meloxicam administration prevented all deleterious effects of LPS on arterial pressures (Figure 2A–C), and attenuated those seen on CVP (Figure 2D), although the rebound effect in DAP and MAP was also observed (Figure 2B,C). SVR also increased in the meloxicam group, but this change was of a lower magnitude compared to the control group (Figure 3D).

### 3.5. Effect of Experimentally Induced Endotoxemia and Meloxicam Administration on Cardiac Function

LPS infusion affected most of the evaluated cardiac parameters (Table 1 and Table 2), with meloxicam ameliorating these deleterious effects (Table 1 and Table 2).

SV, CO and CI remained below the baseline values from 0 min to the end of the experiment (Figure 3A–D) in the control group. Meloxicam administration ameliorated this decrease, with CO and CI returning to baseline at 180 min post-LPS (Figure 3A–D).

## 4. Discussion

This study evaluated the effect of experimentally-induced endotoxemia on hemodynamic parameters and cardiac function in donkeys, and the effect of meloxicam administration on these variables. LPS administration caused a significant increase in cTnI, along with significant decreases in SAP, DAP, MAP, and CVP, as well as marked changes in cardiac function such as diminution of SV and CO. Most of these deleterious effects were attenuated by meloxicam.

In this study, experimentally-induced endotoxemia caused a significant elevation in high-sensitive cTnI concentrations in untreated donkeys. While intravenous digoxin administration did not alter cTnI in donkeys [38], other authors have found significant elevations in response to LPS administration and experimental carbohydrate overload (CHO) [39]. This study is the first in donkeys in determining high-sensitive cTnI with an equid-specific technique validated by IDEXX laboratories. In contrast, previous reports used either a point-of care analyzer or an ELISA method without species-specific validation. High-sensitive cTnI is considered the gold standard for the evaluation of myocardial damage, showing higher sensitivity and lower detection limits than conventional troponin tests [40]. This could explain the differences between our results and previous studies, as demonstrated in horses [41]. Moreover, those previous experiments were focused on later phases of experimentally-induced endotoxemia (with the first sampling made at 6 h post-LPS) and used a lower endotoxin dosage [17,39]. Since different assays were used in those previous studies, a lack of sufficient sensitivity to detect minor changes in cTnI concentrations should also be considered. Additionally, miniature donkeys were included in these previous studies, and a breed effect cannot be discarded. In horses, LPS infusion also induced an increase in blood cTnI concentrations, although this finding was earlier (beginning at 60 min post-LPS) [21]. Meloxicam administration prevented the LPS-induced cTnI increase. Previous studies in donkeys evaluating dexamethasone (in CHO) and flunixin meglumine (in experimentally-induced endotoxemia) also found this effect [17,39], although both drugs had a later onset of their protective effects (12 h) compared to our study (from baseline). Our findings are in accordance with other reports in mice and chickens, where meloxicam has been proven to avoid cardiotoxicity, secondary to LPS or doxorubicin [42,43].

The cTnI elevation observed in this study could be explained by different mechanisms. First, this increase followed the rise in plasma TNFα and IL-1β concentrations after LPS infusion previously reported in donkeys by our research group [18], which could point to a direct effect of these cytokines on the myocardium. However, cytokine levels also increased moderately in the meloxicam group in that previous study, whereas cTnI did not in our results. Severe tachycardia, which was a prominent and early change in non-treated donkeys with experimentally induced endotoxemia [18], also provokes direct myocardial damage, due to reduced oxygen delivery secondary to reduced diastolic time [44]. However, plasma lactate concentrations increased in both groups [18]. Hypotension was observed in the control group after LPS infusion, which could be linked to a lower diastolic filling (preload) and CVP, leading to myocardial damage. Since these mechanisms could only partially justify these findings, additional factors must be involved; for example, the direct damage from LPS on myocardial cells, among others.

The mean cTnI concentration observed in healthy donkeys in this study was in consonance with those previously reported in healthy horses using a high-sensitive validated test and technique [21]. In this sense, a similar cut-off limit, as previously reported in healthy horses (<0.05 ng/mL), could be proposed in healthy donkeys to diagnose cardiac diseases. It is important to remark that most donkeys included in this study were female; however, it has been previously demonstrated that gender does not influence serum cTnI concentrations in horses [45].

No previous studies evaluating the effect of SIRS on hemodynamic parameters in donkeys are available. Donkeys responded to LPS infusion showing hypotension and decreased CVP. Similar findings have been previously reported in horses with experimentally induced endotoxemia [22,23,36,46], as well as horses with colic and different shock degrees [47]. Untreated donkeys showed a rebound in MAP, which was also observed in the meloxicam group, despite this group not developing previous hypotension. Therefore, LPS infusion could have led to the release of vasoconstrictive mediators such as thromboxane, prostaglandin F2, vasopressin or angiotensin II [48,49], independently of arterial pressure changes, leading to the SVR increase observed in both groups. This mechanism could have also been triggered by the CVP decrease observed in both groups, to avoid cardiac collapse. Although the response of these mediators to SIRS have not been studied in donkeys, LPS-challenged horses showed an increase in plasma serotonin and thromboxane B2 concentrations [50]. In addition, a SVR increase was also observed in anesthetized horses after endotoxin administration [46]. On the other hand, an increase in the arterial blood pressure was observed in LPS-challenged conscious standing horses, due to peripheral vasoconstriction and increased peripheral resistance [21], and in anesthetized horses under various surgical procedures and endotoxin administration [46]. Another study in horses did not observe arterial pressure changes after LPS administration [51]. Further studies measuring vasoconstrictive mediators in donkeys are compelling.

While the invasive blood pressure (IBP) is a more sensitive technique compared to NIBP, and a catheter can be placed in conscious animals in the facial or transverse arteries to obtain IBP [52], this option was discarded due to invasiveness, ease and speed for monitoring preparation and to avoid animal discomfort and related movements which could have caused catheter dislodging. Although NIBP has been demonstrated to be a reliable technique to evaluate blood pressure both in standing and anesthetized horses and foals, it is less accurate than IBP in severe cases of hypotension or hypertension, mostly in standing horses [53,54]. Thus, this issue must be taken into consideration.

Cardiac ultrasound parameters have been previously described in several breeds of healthy standard and miniature donkeys [34,55,56,57,58,59,60], but this study is the first one reporting the effect of experimentally induced endotoxemia on echocardiographic parameters in donkeys, specifically in Andalusian breed donkeys. Baseline values for most parameters (both measured and calculated) were within ranges previously described in healthy donkeys [55,58]. However, higher values were observed in this study for parameters related to area and length, which could be explained by donkeys included in this study showing higher mean body weight and LV mass, compared to previous reports [34,55,57,58]. Our baseline results for these size-dependent parameters were indeed closer to previous reports for horses [61,62]. The effect of body weight and breed on echocardiographic measurements have been previously demonstrated in horses [63]. Many of the echocardiographic parameters evaluated (EMS, LVARd, LVARs, LVLRd, LVLRs, LVMA FC/EMS ratio, LVMAFC, MWT_A_FC, MWT_A_FC/EMS ratio, MWT_A_d, MWT_A_s, and RWT_A_d) have not been previously studied in donkeys and cannot be compared, although results were close to those reported in healthy horses [32,61].

Experimentally induced endotoxemia caused a decrease in LVIA, LVEA, LVAR and LVMA, both in the systole and diastole, as well as in FWTIVS, FWTLVFW, LVFAC, LVMAFC and MWTT_A_FC. These findings could explain the decrease in SV, CO and CI. Similar results have been observed in horses with experimentally induced endotoxemia [36] or with naturally occurring SIRS secondary to colic [47,64]. In our study, FS was unchanged, which mimics previous reports in horses with colic [65].

Contrary results have also been reported in experimentally induced endotoxic anesthetized horses, showing a decrease in SVR and increased CO [22,23]. These discrepancies among studies could be attributed to differences in LPS dose, LPS administration protocol (bolus versus infusion), experiments using conscious versus anesthetized animals, effects of inhalation anesthetic agents, vasopressor and/or inotropic drugs during anesthesia maintenance, etc. Furthermore, SV and CO were calculated using the bullet method. Despite this method having a good agreement with the lithium-dilution method (the gold standard) in horses [66], comparisons between calculated results using different methods should be made carefully. Further studies using lithium dilution and different methods of calculation are needed.

Several anti-inflammatory drugs have been tested in horses with natural SIRS or experimentally induced endotoxemia [27,29,67,68], and even flunixin meglumine and dexamethasone have been studied in donkeys with experimentally induced endotoxemia and SIRS, secondary to CHO, respectively [17,39]. However, this study is the first one evaluating the effect of intravenous meloxicam administration on cardiac function in experimentally induced endotoxemic donkeys. Meloxicam treatment partially or totally prevented the decrease in LVIA, LVEA, LVAR, LVMA, FWTIVS, FWTLVFW, LVFAC, LVMAFC and MWTT_A_FC. These findings demonstrate a beneficial therapeutical effect of intravenous meloxicam on cardiac (ventricular) derangements in experimentally induced endotoxemic donkeys.

Cyclooxygenase (COX)-2-selective (meloxicam) and non-selective (flunixin meglumine) NSAIDs have been proven to be useful in the treatment of SIRS in donkeys [17,18]. Although pharmacodynamic studies and reports about short- and long-term side effects of meloxicam have not available in donkeys, meloxicam has a short half-life, lower mean residence time, and faster clearance in this species [30]. Thus, this drug can be used in donkeys with SIRS, showing a rapid effect and avoiding any long-lasting toxicity. Moreover, even higher doses (1.2 mg/kg/PO) of this drug in donkeys have been reported to show satisfactory effects [69], but toxicity was not evaluated in this study.

In order to avoid any operator bias, only one clinician performed all the ultrasound examinations, while a different blinded clinician conducted the measurements. Nonetheless, it has been demonstrated that the intra-operator, intra-observer and inter-operator variability of transthoracic echocardiographic measurements is low, with a good repeatability in donkeys, horses and foals [58,70,71].

## 5. Conclusions

Acute experimentally induced endotoxemia provokes marked hemodynamic and cardiac dysfunction in donkeys, causing a significant increase in cTnI, hypotension, decreased CVP, SV and CO, and changes in cardiac ultrasound parameters. Since meloxicam administration was able to prevent and/or alleviate most of these deleterious effects, this drug could be an effective therapeutic option in donkeys with hemodynamic derangements and cardiac dysfunction secondary to SIRS. Additional studies comparing meloxicam with other non-steroidal anti-inflammatories (NSAIDs) or alternative drugs in donkeys with SIRS are needed.

## Figures and Tables

**Table 1:** Results for cardiac parameters measured on transthoracic echocardiography from right parasternal plane, both short and long axis, in M- and B-modes.

		Time (Minutes)								
Parameter	Group	−30	0	30	60	90	120	150	180	240
EMS (s)	Control	0.4 ± 0.01	0.4 ± 0.01	0.3 ± 0.01 ^a^	0.3 ± 0.02 ^a^	0.4 ± 0.03 ^a^	0.3 ± 0.04	0.4 ± 0.04	0.4 ± 0.02	0.4 ± 0.05
	Meloxi	0.4 ± 0.02	0.4 ± 0.00	0.4 ± 0.01	0.4 ± 0.01 ^b^	0.4 ± 0.01	0.4 ± 0.01	0.4 ± 0.02	0.4 ± 0.03	0.4 ± 0.05
IVSd (cm)	Control	2.2 ± 0.2	2.4 ± 0.1	2.5 ± 0.2	2.6 ± 0.3	2.4 ± 0.3	2.1 ± 0.4	2.2 ± 0.3	2.1 ± 0.3	2.1 ± 0.4
	Meloxi	2.1 ± 0.1	2.2 ± 0.3	2.5 ± 0.3	2.4 ± 0.2	2.5 ± 0.1 ^a^	2.5 ± 0.3	2.7 ± 0.3	2.4 ± 0.1 ^a^	2.9 ± 0.4
IVSs (cm)	Control	3.6 ± 0.2	3.6 ± 0.3	3.7 ± 0.3	3.7 ± 0.4	3.7 ± 0.5	3.1 ± 0.5	2.8 ± 0.6	3.1 ± 0.5	3.1 ± 0.6
	Meloxi	3.4 ± 0.2	3.5 ± 0.3	3.7 ± 0.2	3.8 ± 0.2	3.7 ± 0.2 ^a^	3.7 ± 0.2	3.5 ± 0.3	3.4 ± 0.3	3.9 ± 0.2
LVFWd (cm)	Control	1.9 ± 0.2	2.1 ± 0.1 ^a^	2.2 ± 0.2 ^a^	2.1 ± 0.1	2.1 ± 0.2	1.8 ± 0.3	1.6 ± 0.4	1.7 ± 0.4	1.5 ± 0.5
	Meloxi	2.0 ± 0.1	2.1 ± 0.2	1.9 ± 0.2	1.9 ± 0.1	1.9 ± 0.1	1.9 ± 0.2	2.0 ± 0.2	1.9 ± 0.2	2.2 ± 0.3
LVFWs (cm)	Control	3.1 ± 0.3	3.1 ± 0.3	3.1 ± 0.2	3.1 ± 0.1	3.0 ± 0.3	2.9 ± 0.3	2.3 ± 0.5	2.7 ± 0.4	2.9 ± 0.5
	Meloxi	3.1 ± 0.2	2.9 ± 0.3	3.0 ± 0.3	3.2 ± 0.3	3.1 ± 0.2	4.3 ± 1.0	2.9 ± 0.2	3.0 ± 0.1	3.6 ± 0.3
LVIDd (cm)	Control	7.9 ± 0.7	7.8 ± 0.7	7.6 ± 0.7	7.3 ± 0.9 ^a^	7.5 ± 1.1 ^a^	6.5 ± 0.6	7.0 ± 0.5	6.9 ± 0.5	6.9 ± 0.6
	Meloxi	8.0 ± 0.4	7.5 ± 0.4	8.0 ± 1.1	7.4 ± 0.4	7.8 ± 0.6	7.7 ± 0.5	7.8 ± 0.4	7.9 ± 0.9	8.3 ± 0.5
LVIDs (cm)	Control	4.5 ± 0.3	4.7 ± 0.3	4.3 ± 0.5	4.1 ± 0.8	4.5 ± 0.5	4.3 ± 0.8	4.5 ± 0.3	3.9 ± 0.6	3.9 ± 0.4
	Meloxi	4.6 ± 0.2	4.2 ± 0.3	4.9 ± 0.9	3.8 ± 0.3	4.1 ± 0.4	4.3 ± 0.5	4.8 ± 0.3	4.5 ± 0.3	4.8 ± 0.5
LVEAd (cm^2^)	Control	114.6 ± 11.9	110.5 ± 18.1	108.2 ± 16.7	105.6 ± 18.9	113.3 ± 46.7	94.2 ± 15.6	94.7 ± 16.8	91.3 ± 18.4	92.7 ± 12.3
	Meloxi	122.5 ± 12.8	110.1 ± 17.6	119.8 ± 16.2	111.3 ± 16.5	115.9 ± 13.2	113.8 ± 10.9	110.8 ± 14.4	109.2 ± 16.2	114.3 ± 14.0 ^b^
LVEAs (cm^2^)	Control	90.1 ± 12.9	91.7 ± 13.0 ^a^	86.8 ± 11.8	83.8 ± 15.3	85.9 ± 28.1	83.4 ± 15.6	81.4 ± 12.2	86.2 ± 15.6	85.7 ± 13.7
	Meloxi	95.3 ± 10.6	85.8 ± 13.1 ^a^	90.8 ± 9.7 ^a^	90.9 ± 11.9	92.5 ± 10.3	91.4 ± 9.8	88.8 ± 8.9 ^a^	89.1 ± 13.4	96.7 ± 5.2
LVIAd (cm^2^)	Control	44.8 ± 3.0	40.3 ± 6.8	37.3 ± 5.5 ^a^	33.7 ± 7.1	36.7 ± 11.3	36.0 ± 10.8 ^a^	41.6 ± 2.4	41.8 ± 3.1	37.5 ± 9.3
	Meloxi	50.1 ± 6.0	43.2 ± 6.4	45.6 ± 7.4	41.3 ± 7.5 ^a^	42.6 ± 4.9	43.6 ± 5.0 ^b^	42.6 ± 6.8	43.1 ± 7.2	50.6 ± 4.7 ^b^
LVIAs (cm^2^)	Control	10.7 ± 2.3	12.8 ± 2.2	9.8 ± 2.3 ^a^	8.9 ± 2.4	8.7 ± 4.8	9.8 ± 3.1	11.2 ± 2.5	9.2 ± 3.1 ^a^	8.1 ± 2.7
	Meloxi	13.1 ± 2.3	10.3 ± 2.2	12.1 ± 2.6	8.8 ± 3.1 ^a^	9.9 ± 1.2	12.2 ± 2.5	12.7 ± 2.1	15.3 ± 3.0 ^a,b^	14.8 ± 3.1 ^b^
LVARd (cm^2^)	Control	110.3 ± 17.4	103.9 ± 15.9	101.8 ± 18.1	94.5 ± 18.7	99.1 ± 21.6	84.8 ± 20.7	85.5 ± 14.3	94.8 ± 15.1	84.0 ± 17.6
	Meloxi	110.6 ± 12.6	101.7 ± 21.1	100.7 ± 11.6	99.4 ± 11.6	95.9 ± 9.3	101.6 ± 13.7	102.3 ± 12.3	96.4 ± 16.5	119.3 ± 19.2
LVARs (cm^2^)	Control	36.7 ± 2.8	38.4 ± 2.9	34.8 ± 1.5	34.7 ± 2.3	32.5 ± 2.0	30.6 ± 4.6	34.9 ± 3.9	38.5 ± 6.9	39.0 ± 7.4
	Meloxi	39.2 ± 3.7	34.1 ± 4.2	41.3 ± 2.5	37.5 ± 4.7	31.6 ± 4.1	38.9 ± 4.9	43.2 ± 6.3	38.1 ± 7.1	40.1 ± 6.8
LVLRd (cm)	Control	16.4 ± 1.6	15.9 ± 1.2	15.5 ± 1.0	15.5 ± 1.6	15.6 ± 1.7	13.4 ± 1.5	14.2 ± 1.3	15.9 ± 1.6	14.6 ± 1.1
	Meloxi	15.5 ± 1.1	15.2 ± 0.5	15.3 ± 1.1	15.1 ± 1.1	14.9 ± 0.8	15.1 ± 1.5	15.6 ± 1.4	14.9 ± 1.5	15.5 ± 1.2
LVLRs (cm)	Control	10.1 ± 0.8	10.4 ± 1.1	10.2 ± 0.6	9.9 ± 0.5	9.9 ± 0.3	9.3 ± 0.4	10.1 ± 0.5	11.9 ± 0.9	10.2 ± 0.3
	Meloxi	10.2 ± 0.6	9.7 ± 0.4	10.5 ± 0.2	10.3 ± 0.5	9.4 ± 0.4	10.3 ± 0.8	10.5 ± 0.7	10.2 ± 1.0	10.5 ± 0.3

Data are expressed as mean ± standard error of the mean (SEM). EMS, electromechanical systole; IVSd, interventricular septal thickness at end-diastole; IVSs, interventricular septal thickness at peak systole; LVFWd, left-ventricular free wall at end-diastole; LVFWs, left-ventricular free wall at peak systole; LVIDd, left-ventricular internal diameter at end-diastole; LVIDs, left-ventricular internal diameter at peak systole; LVEAd, left-ventricular external area at end-diastole; LVEAs, left-ventricular external area at peak systole; LVIAd, left-ventricular internal area at end-diastole; LVIAs, left-ventricular internal area at peak systole; LVARd, left-ventricular area at end-diastole; LVARs, left-ventricular area at peak systole; LVLRd, left-ventricular length at end-diastole; LVLRs, left-ventricular length at peak systole; Meloxi, meloxicam treated group. ^a^ *p* < 0.05 vs. −30 time-point. ^b^ *p* < 0.05 vs. similar time-point in control group.

**Table 2:** Results of cardiac parameters calculated from measurements performed on transthoracic echocardiography from right parasternal plane, both short and long axis, in M- and B-modes.

		Time (Minutes)								
Parameter	Group	−30	0	30	60	90	120	150	180	240
EF (%)	Control	81 ± 4	77 ± 8 ^a^	81 ± 9	82 ±	82 ± 4	78 ± 6	72 ± 1 ^a^	83 ± 5 ^a^	79 ± 7 ^a^
	Meloxi	81 ± 1	83 ± 2 ^b^	78 ± 4	86 ± 3	84 ± 2	83 ± 3	76 ± 2	78 ± 2	81 ± 2
FS (%)	Control	42.6 ± 0.4	39.5 ± 0.7 ^a^	42.8 ± 0.9	43.9 ± 4.0	43.9 ± 3.9	40.9 ± 6.2	35.3 ± 0.1	45.4 ± 0.3	41.5 ± 0.5
	Meloxi	43.1 ± 1.2	44.9 ± 2.4 ^b^	40.4 ± 3.5	49.3 ± 3.5	46.9 ± 2.8	45.3 ± 3.3	38.4 ± 2.1	40.4 ± 1.9	43.1 ± 2.5
FWTIVS (%)	Control	51.3 ± 11.3	39.2 ± 7.7	38.4 ± 9.8	32.0 ± 10.7	40.1 ± 15.9	35.7 ± 22.6	21.7 ± 12.3	34.0 ± 16.7	33.8 ± 21.8
	Meloxi	72.3 ± 6.1	59.1 ± 8.0	52.1 ± 7.5	62.4 ± 9.1 ^b^	51.1 ± 6.2 ^a^	48.8 ± 7.4 ^a^	33.4 ± 10.8 ^a^	43.4 ± 8.7 ^a^	40.7 ± 15.7
FWTLVFW (%)	Control	64.9 ± 8.3	47.2 ± 10.6 ^a^	45.9 ± 9.6 ^a^	47.1 ± 5.4	51.8 ± 12.4	69.6 ± 4.6	50.8 ± 8.1 ^a^	69.1 ± 23.5	67.3 ± 14.7
	Meloxi	56.8 ± 4.9	46.2 ± 4.0	61.9 ± 15.7	67.8 ± 3.3 ^b^	65.5 ± 4.6	52.5 ± 11.2	44.7 ± 7.5	58.8 ± 16.8	68.7 ± 11.8
LVFAC (%)	Control	61.6 ± 14.4	52.7 ± 12.4	59.0 ± 13.9	58.9 ± 14.0	60.1 ± 19.0 ^a^	51.7 ± 23.8 ^a^	46.0 ± 20.7	47.8 ± 21.9	51.2 ± 23.7
	Meloxi	74.5 ± 1.9	76.9 ± 2.1	73.9 ± 5.3	81.1 ± 3.6 ^a^	75.9 ± 2.7	72.7 ± 3.1	68.1 ± 1.9 ^a^	64.9 ± 3.3 ^a^	71.5 ± 4.0
LVMAFC/ EMS ratio	Control	26.6 ± 9.1	22.9 ± 10.9	18.9 ± 11.8	15.6 ± 8.1 ^a^	30.4 ± 20.2	29.2 ± 14.6	19.7 ± 12.9	17.6 ± 10.6	26.6 ± 12.2
	Meloxi	30.6 ± 8.2	35.6 ± 10.8	20.6 ± 8.6	50.8 ± 13.5 ^b^	38.7 ± 7.8	30.4 ± 5.5	16.3 ± 4.5	27.6 ± 12.2	24.5 ± 12.2
LVMAd (cm^2^)	Control	53.6 ± 13.5	56.1 ± 14.1	54.9 ± 14.0	54.3 ± 14.2	52.6 ± 20.4	35.0 ± 17.4	37.4 ± 17.6	38.3 ± 17.9	36.1 ± 17.2
	Meloxi	72.4 ± 7.3	66.9 ± 11.2	74.2 ± 9.2	69.9 ± 9.7	73.3 ± 8.8 ^b^	70.2 ± 6.5	68.2 ± 7.7	66.1 ± 8.9	83.7 ± 3.2 ^b^
LVMAs (cm^2^)	Control	75.5 ± 8.5	74.5 ± 9.0	73.4 ± 7.6	72.2 ± 9.9	74.1 ± 13.8	68.5 ± 9.9	60.7 ± 0.5	60.8 ± 3.8	59.9 ± 6.2
	Meloxi	82.2 ± 8.7	75.4 ± 10.9	78.7 ± 7.9	82.0 ± 9.5	82.5 ± 10.2	79.2 ± 8.3	76.1 ± 7.4 ^a^	73.8 ± 10.5 ^a^	91.9 ± 4.1 ^a,b^
LVMAFC (%)	Control	13.4 ± 3.8	6.7 ± 5.4	8.4 ± 4.9	6.3 ± 3.4 ^a^	16.6 ± 11.0	15.5 ± 5.9	11.2 ± 7.5	9.5 ± 5.5	14.1 ± 3.4
	Meloxi	13.4 ± 4.1	14.5 ± 4.5	7.6 ± 3.3	19.8 ± 5.7	12.6 ± 4.1	12.3 ± 2.4	6.9 ± 2.2	11.8 ± 4.9	9.9 ± 5.1
LVmass (g)	Control	1320 ± 287	1420 ± 243	1456 ± 328	1392 ± 312	1338 ± 397	866 ± 129	976 ± 128	927 ± 78	826 ± 157
	Meloxi	1370 ± 204	1351 ± 311	1589 ± 418	1305 ± 209	1463 ± 268	1477 ± 297	1603 ± 285	1532 ± 468	1716 ± 233 ^b^
MWT_A_FC (%)	Control	56.1 ± 4.2	46.4 ± 7.8	44.1 ± 7.6	40.3 ± 6.2	51.7 ± 11.4	52.4 ± 12.9	42.1 ± 9.3 ^a^	42.9 ± 7.9	49.9 ± 1.4
	Meloxi	54.5 ± 3.4	57.4 ± 6.5 b	45.8 ± 3.2	64.8 ± 9.3 ^b^	52.0 ± 5.8	49.7 ± 3.9	40.3 ± 3.4	42.9 ± 7.2	45.5 ± 4.6
MWT_A_FC/ EMS ratio	Control	136.7 ± 9.6	129.2 ± 16.9	123.7 ± 19.4	119.9 ± 16.3	140.4 ± 24.5	145.5 ± 17.2	111.8 ± 12.0	116.2 ± 13.2	140.6 ± 14.7
	Meloxi	128.0 ± 5.3	141.3 ± 15.4 ^b^	123.0 ± 10.2	138.1 ± 21.9	140.9 ± 11.0	124.5 ± 11.0	97.5 ± 5.2	103.7 ± 15.0	118.2 ± 15.9
MWT_A_d (cm)	Control	2.2 ± 0.1	2.3 ± 0.1	2.3 ± 0.1	2.4 ± 0.1	2.4 ± 0.3	2.1 ± 0.3	2.1 ± 0.2	2.1 ± 0.2	2.1 ± 0.2
	Meloxi	2.2 ± 0.1	2.2 ± 0.2	2.3 ± 0.1	2.3 ± 0.1	2.4 ± 0.1	2.3 ± 0.1	2.2 ± 0.1	2.2 ± 0.1	2.5 ± 0.1
MWT_A_s (cm)	Control	3.4 ± 0.2	3.2 ± 0.2	3.3 ± 0.2	3.4 ± 0.3	3.5 ± 0.2	3.1 ± 0.2	2.9 ± 0.1	2.9 ± 0.1	3.1 ± 0.3
	Meloxi	3.5 ± 0.2	3.4 ± 0.2	3.4 ± 0.2	3.7 ± 0.2	3.6 ± 0.3	3.4 ± 0.2	3.3 ± 0.2 ^a^	3.1 ± 0.2 ^a^	3.7 ± 0.2
MWTd (cm)	Control	2.0 ± 0.1	2.2 ± 0.1	2.3 ± 0.2 ^a^	2.3 ± 0.2 ^a^	2.2 ± 0.2	1.9 ± 0.1	1.9 ± 0.1	1.9 ± 0.1 ^a^	1.8 ± 0.2
	Meloxi	2.0 ± 0.1	2.1 ± 0.2	2.2 ± 0.2	2.1 ± 0.2	2.2 ± 0.1	2.2 ± 0.2	2.3 ± 0.2	2.2 ± 0.2	2.5 ± 0.3
MWTs (cm)	Control	3.3 ± 0.2	3.3 ± 0.2	3.3 ± 0.2	3.2 ± 0.2	3.3 ± 0.2	2.9 ± 0.1	2.6 ± 0.1	3.0 ± 0.1	2.7 ± 0.1
	Meloxi	3.3 ± 0.2	3.2 ± 0.3	3.4 ± 0.3	3.5 ± 0.2	3.4 ± 0.2	3.9 ± 0.5	3.2 ± 0.2	3.2 ± 0.2	3.8 ± 0.2 ^b^
RWT_A_d	Control	0.6 ± 0.0	0.7 ± 0.1	0.7 ± 0.0	0.7 ± 0.1	0.7 ± 0.1	0.7 ± 0.1	0.6 ± 0.1	0.6 ± 0.1	0.7 ± 0.1
	Meloxi	0.6 ± 0.0	0.6 ± 0.0	0.6 ± 0.10	0.6 ± 0.1	0.6 ± 0.1 ^a^	0.6 ± 0.0 ^a^	0.6 ± 0.0 ^a^	0.6 ± 0.0 ^a^	0.6 ± 0.1 ^a^
RWTd	Control	0.5 ± 0.00	0.6 ± 0.02	0.6 ± 0.02 ^a^	0.6 ± 0.03 ^a^	0.6 ± 0.02	0.6 ± 0.04	0.5 ± 0.04	0.5 ± 0.01	0.5 ± 0.03
	Meloxi	0.5 ± 0.02	0.6 ± 0.04	0.6 ± 0.07	0.6 ± 0.04	0.6 ± 0.02	0.6 ± 0.06	0.6 ± 0.07	0.5 ± 0.03	0.6 ± 0.10

Data are expressed as mean ± standard error of the mean (SEM). EF, ejection fraction; FS, fractional shortening; FWTIVS, fractional wall thickening of the interventricular septum; FWTLVFW, fractional wall thickening of the LV free wall; LVFAC, left ventricular fractional area change; LVMA FC/EMS ratio, fractional change in left ventricular myocardial area/electromechanical systole ratio; LVMAd, left-ventricular myocardial area at end-diastole; LVMAs, left-ventricular myocardial area at peak systole; LVMAFC, fractional change in left-ventricular myocardial area; LVmass, left ventricular mass; Meloxi, meloxicam-treated group; MWTAFC, fractional change in mean wall thickness; MWTAFC/EMS ratio, mean wall thickness at end-systole/electromechanical systole ratio; MWTAd, mean wall thickness at end-diastole in SAX D-mode; MWTAs, mean wall thickness at peak systole in SAX D-mode; MWTd, mean wall thickness at end-diastole in SAX M-mode; MWTs, mean wall thickness at peak systole in SAX M-mode; RWTAd, relative wall thickness at end-diastole in SAX D-mode; RWTd, relative wall thickness diastole in SAX M-mode. ^a^ *p* < 0.05 vs. −30 time-point. ^b^ *p* < 0.05 vs. similar time-point in control group.

## Data Availability

Data are available upon request from the corresponding authors.

## Supplementary Materials

The following supporting information can be downloaded at: https://www.mdpi.com/article/10.3390/ani14243660/s1, Table S1: Transthoracic echocardiography measurements performed from a right parasternal plane, both in a short and long axis, in M- and D-mode. Table S2: Calculations obtained from the transthoracic echocardiography measurement performed from the right parasternal plane, both in short and long axis, in M- and B-mode.
