# Supplementary material for: Hemodynamic Response to Lipopolysaccharide Infusion and Effect of Meloxicam Administration on Cardiac Function in Donkeys
Source: Animals (Basel). 2024 Dec 18;14(24):3660. doi: 10.3390/ani14243660 (PMC11672460; doi:10.3390/ani14243660)
Supplement: Supplementary file 1 [file animals-14-03660-s001.zip › animals-3348008-supplementary.pdf]

Table S1. Transthoracic echocardiography measurements performed from a right parasternal plane, both in a short- and long axis, in M- and D-mode.

Mode	View	Parameter	Definition
M-mode	SAX	EMS	Electromechanical systole
		IVSd	interventricular septal thickness at end-diastole
		IVSs	interventricular septal thickness at peak systole
		LVFWd	LV free wall at end-diastole
		LVFWs	LV free wall at peak systole
		LVIDd	LV internal diameter at end-diastole
		LVIDs	LV internal diameter at peak systole
D-mode	SAX	LVEAd	LV external area at end-diastole
		LVEAs	LV external area at peak systole
		LVIAd	LV internal area at end-diastole
		LVIAs	LV internal area at peak systole
	LAX	LVARd	LV area at end-diastole
		LVARs	LV area at peak systole
		LVLRd	LV length at end-diastole
		LVLRs	LV length at peak systole

LAX, long-axis 4-chamber view; LV, left ventricle; SAX, short-axis view of the left ventricle.

Table S2. Calculations obtained from the transthoracic echocardiography measurement performed from the right parasternal plane, both in short- and long axis and in M- and B-mode.

Parameter	Definition	Calculation
CI	Cardiac index	CO / BSA
CObullet	Cardiac output bullet method	HR x SVbullet
EF	Ejection fraction	[(LVIVd^3 – LVIVs^3) / LVIVd^3] x 100
FS	Fractional shortening	[(LVIDd - LVIDs) / LVIDd] x 100
FWTIVS	Fractional wall thickening of the interventricular septum	IVSs-IVSd/IVSd x 100
FWTLVFW	Fractional wall thickening of the LV free wall	LVFWs-LVFWd/LVFWd x 100
LVFAC	Left ventricular fractional area change	(LVIAd - LVIAs) / LVIAd x 100
LVMA FC/ EMS ratio	Fractional change in left ventricular myocardial area / electromechanical systole ratio	LVMA FC / EMS
LVMAd	Left-ventricular myocardial area at end-diastole	LVEAd - LVIAd
LVMAs	Left-ventricular myocardial area at peak systole	LVEAs - LVIAs
LVMA FC	Fractional change in left-ventricular myocardial area	(LVMAs - LVMAd) / LVMAd x 100
LVmass	Left ventricular mass	1.04 x (LVIDd+LVFWd+IVSd)^3-LVIDd^3)-13.6
MWT$_A$d	Mean wall thickness at end-diastole in SAX D-mode	$\sqrt{}$ (LVEAd / π) - $\sqrt{}$ (LVIAd / π)
MWT$_{AS}$	Mean wall thickness at peak systole in SAX D-mode	$\sqrt{}$ (LVEAs / π) - $\sqrt{}$ (LVIAs / π)
MWT$_A$FC	Fractional change in mean wall thickness	(MWT$_{AS}$ - MWT$_A$d) / MWT$_A$d x 100
MWT$_A$FC / EMS ratio	Mean wall thickness at end systole / electromechanical systole ratio	MWT$_A$FC / EMS
MWTd	Mean wall thickness at end-diastole in SAX M-mode	(IVSd + LVFWd) / 2
MWTs	Mean wall thickness at peak systole in SAX M-mode	(IVSs + LVFWs) / 2
RWT$_A$d	Relative wall thickness at end-diastole in SAX D-mode	[$\sqrt{}$ (LVEAd / π) - $\sqrt{}$ (LVIAd / π)] / $\sqrt{}$ (LVIAd / π)
RWTd	Relative wall thickness diastole in SAX M-mode	(IVSd + LVFWd) / LVIDd
SVbullet	Stroke volume bullet method	(5/6 x LVIAd x LVLRd) – (5/6 x LVIAs x LVLRs)

BSA, body surface area; LAX, long-axis 4-chamber view; LV, left ventricle; SAX, short-axis view of the left ventricle.
